# Case Report: Resolution of complete heart block following vitamin D supplementation in a child with Graves disease

**DOI:** 10.3389/fendo.2025.1680344

**Published:** 2026-01-09

**Authors:** Sofya Ilmer, Parissa Salemi

**Affiliations:** 1 Northwell Health, New Hyde Park, NY, United States; 2 Division of Pediatric Endocrinology, Department of Pediatrics, Cohen Children’s Medical Center, New Hyde Park, NY, United States

**Keywords:** atrioventricular block, autoimmune diseases, complete heart block, Graves disease, hyperthyroidism, pediatrics, vitamin D

## Abstract

This case report describes an 11-year-old male with Graves disease and pre-existing Mobitz type 1 second-degree atrioventricular (AV) block that progressed to complete heart block (CHB) one month prior to his diagnosis of Graves disease. He presented at age 9 years (January 2023) with weight loss, heat intolerance, and excessive sweating. Laboratory testing revealed: thyroid stimulating hormone (TSH) <0.01 uIU/mL, free thyroxine (FT4) 4.8 ng/dL, total thyroxine (T4) 16.7 µg/dL, and total triiodothyronine (T3) 334 ng/dL. He was treated with methimazole and was biochemically euthyroid within one month of treatment. Despite becoming euthyroid, the CHB persisted. By April of 2023, his cardiologist recommended the implantation of a pacemaker, however the family opted for continued monitoring instead. Following his mother’s research into potential benefits of vitamin D in autoimmune thyroid disease, vitamin D3 supplementation 2500 IU daily was initiated in late October 2023. Three months later, his nighttime bradycardia resolved. A 12-lead ECG in April 2024 confirmed the resolution of CHB to first-degree AV block. No additional medications were used to treat his cardiac condition. Patient remained stable with first-degree heart block while euthyroid on low dose methimazole. He continued vitamin D supplementation at 2000 IU daily. Peak TSH receptor antibody (TSHRab) and thyroid-stimulating immunoglobulin (TSI) values decreased following vitamin D3 initiation. Several proposed mechanisms may explain this observation, including vitamin D’s immunomodulatory effects on Graves disease, its cardioprotective properties, and its potential synergistic effect with methimazole in achieving better thyroid control. This case highlights a potential association between vitamin D supplementation and improved AV block in a pediatric patient with Graves disease, warranting further investigation into vitamin D’s role in managing cardiac manifestations of autoimmune thyroid disease.

## Introduction

Graves disease (GD) is an autoimmune disorder characterized by hyperthyroidism due to stimulating antibodies directed against the TSH receptor (TSHRab) which can have significant cardiovascular implications. Hyperthyroidism exerts multiple effects on the cardiovascular system, including an increase in heart rate, myocardial contractility, and cardiac output while decreasing systemic vascular resistance. These changes can predispose patients to various dysrhythmias, including atrial fibrillation and AV block (1).

AV block in the context of hyperthyroidism can be underrecognized, potentially masked by the more common tachyarrhythmias associated with this condition. The clinical course of AV block in hyperthyroidism is variable, ranging from spontaneous resolution with achievement of euthyroid state, to persistent conduction abnormalities requiring intervention (1, 2). Emerging evidence suggests that vitamin D deficiency may contribute to the pathogenesis and severity of Graves disease, with recent studies exploring its immunomodulatory and potential therapeutic roles in this context (3–5). Given the potential link between vitamin D and autoimmune thyroid disease, and its potential cardioprotective effects, this case report explores the impact of vitamin D supplementation on a pediatric patient with Graves disease and complete heart block.

## Case description

**Figure 1:**
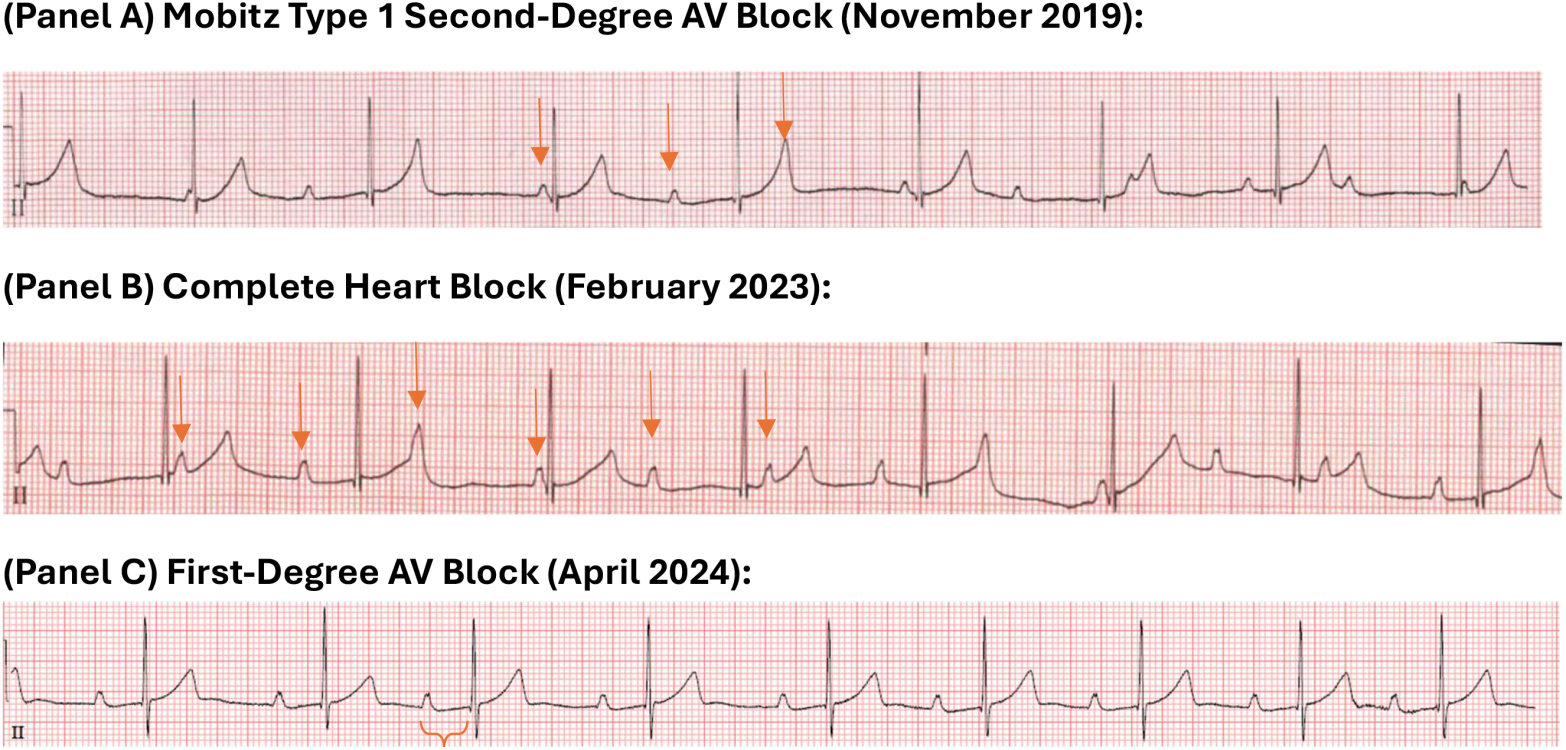
ECG rhythm strips demonstrating evolution of atrioventricular block. Evolution of atrioventricular (AV) block. Rhythm strips are from Lead II **(A)** A tracing from November 2019 demonstrates Mobitz type 1 second-degree AV block. The PR interval progressively lengthens before a non-conducted P-wave occurs. Arrows mark P-waves. **(B)** A tracing from February 2023 shows complete heart block with complete atrioventricular dissociation (atrial rate approx. 75 bpm, ventricular escape rate approx. 50 bpm). Arrows mark P-waves. **(C)** A tracing from April 2024, following vitamin D supplementation, demonstrates resolution to first-degree AV block with stable 1:1 conduction and a fixed, prolonged PR interval of ~310ms. PR interval is marked.

A 9-year-old Asian American male presented in January 2023 with symptoms of GD including weight loss, heat intolerance, excessive sweating, and palpitations. Laboratory testing by his allergist whom he was following for hives, showed a suppressed TSH and FT4 significantly above the normal range which prompted an urgent endocrinology referral. He denied fatigue or weakness. His physical examination was remarkable for a mildly enlarged thyroid gland without nodules or asymmetry, but otherwise normal exam, including no audible murmur, no edema, or any other signs of Graves disease (e.g., exophthalmos, tremors, skin changes). Vital signs were normal with a heart rate of 66 and a blood pressure of 117/76. Confirmatory thyroid function tests revealed: TSH <0.01 uIU/mL, Free T4 4.8 ng/dL, T4 16.7 µg/dL, T3–334 ng/dL. Baseline TSHRab and TSI values were 11.6 IU/L and 18 IU/L respectively (see **Table 1**). Family history is remarkable for GD on both sides of the family including his mother, father and maternal grandmother. Patient has a past medical history significant for Mobitz type 1 second-degree heart block diagnosed when he was six years old (**Figure 1A**). Of note, he completed a primary COVID-19 vaccination series in late 2021. The etiology of his initial atrioventricular block (AVB) was undetermined. An initial cardiologic evaluation showed normal Lyme titers, thyroid function tests, and antinuclear antibodies (ANA). A maternal screen for congenital heart block was also negative, with normal anti-Ro/SSA and anti-La/SSB antibody levels. One month prior to his diagnosis of Graves disease, his cardiac condition progressed to a complete heart block (CHB), as documented by a Holter monitor in December 2022. Upon diagnosis of Graves disease he was treated with methimazole and achieved a biochemically euthyroid state within one month of treatment. Based on ECG and Holter monitoring, although the patient became euthyroid, the CHB persisted (**Figure 1B**). At his visits with cardiology in March and April, his diurnal resting heart rates were 41 and 49 in the setting of CHB and so, in April 2023 his cardiologist recommended the implantation of a pacemaker. The family opted for continued monitoring instead. In late October 2023, the patient’s mother began vitamin D3 supplementation in her son with 2500 IU/day after researching the potential benefits of vitamin D in autoimmune thyroid disease. The dose was decided on based on discussion with a family member’s adult endocrinologist. Baseline vitamin D levels were not obtained prior to the start of supplementation. Three months after vitamin D initiation, in February 2024, the patient’s overnight heart rate, which was previously in the 30–40 beats per minute (bpm) range, normalized to an average of 60–80 bpm. Our team was not aware about the vitamin D supplementation until they returned for follow up and reported improvement in his overnight heart rates. This improvement was subsequently confirmed by electrocardiogram in April 2024, demonstrating resolution of CHB to first-degree AV block (**Figure 1C**). His medications included only methimazole and vitamin D supplementation at that time. At the time of this report, the 12-year-old patient remains stable with first-degree AV block and is euthyroid on methimazole 2.5 mg daily. He continues vitamin D supplementation at 2000 IU/day with minimal missed doses as reported by his mother, with 25-hydroxyvitamin D levels ranging between 63.5-78.1 ng/mL and his calcium between 10.0-10.7 mg/dL (corrected 9.6-10.0 mg/dL based on albumin levels). This decreased vitamin D dosage was recommended by our team when his vitamin D levels were 78.1 ng/mL in November 2024. Over time the peak TSHRab and TSI values notably decreased (see **Table 1**).

**Table 1:** Thyroid hormone, vitamin D and antibody trends.

Laboratory test and reference range	Date										
	Jan-23*	Feb-23	Mar-23	May-23	Jul-23	Nov-23**	Apr-24	June-24	Nov-24	Feb-25	Jul-25
TSH (ref 0.6-4.8 uIU/mL)	<0.01	<0.01	0.09	0.01	3.62	3.00	4.86	3.32	3.28	3.73	4.73
Free T4 (ref 0.9-1.8 ng/dL)	4.8	1.2	1.1	1.7	1.2	1.4	1.4	1.4	1.4	1.4	1.4
T4 (ref 4.6-12.0 µg/dL)	16.7	6.7	6.6	–	–	–	7.4	8.0	7.4	8.8	7.0
T3 (ref 80–200 ng/dL)	334	120	117	137	125	122	121	147	116	144	119
TSHRAb (ref <1.75 IU/L)	18	–	–	–	–	–	–	–	2.54	2.35	2.00
TSI (ref <0.55 IU/L)	11.6	12.3	9.54	–	–	–	4.81	3.83	3.06	–	1.76
25-OH Vitamin D (ref 30–80 ng/mL)	–	–	–	–	–	–	–	–	78.1	70.8	63.5
Calcium (8.4-10.5 mg/dL)	10.7	–	–	–	–	–	–	–	10.6	10.0	10.5
Corrected calcium (mg/dL)	10.2	–	–	–	–	–	–	–	10.0	9.6	10.1
Methimazole dose (mg/kg/d)	0.361	0.337	0.080	0.078	0.094	0.091	0.086	0.082	0.082	0.080	0.078

*January 2023 - diagnosis of GD and initiation of methimazole; **Nov 2023–2 weeks after starting vitamin D. [-] level not obtained

## Discussion

This case report presents a potential association between vitamin D supplementation and the resolution of complete heart block in a pediatric patient with GD, raising questions about the interplay between vitamin D, autoimmunity, and cardiac function. The occurrence of CHB in pediatric GD is unusual, adding to the uniqueness of this case and the novelty of using vitamin D in this context.

The potential association between vitamin D supplementation and the improvement in AV block in patients with GD is an emerging area of interest. Vitamin D is known to have significant immunomodulatory effects, which may influence the course of autoimmune thyroid diseases such as GD. Studies have shown that vitamin D deficiency is common in patients with GD and is associated with higher titers of thyroid autoantibodies, suggesting a potential role in the pathogenesis and progression of the disease (6–8).

TSH receptors (TSHR) are expressed not only on thyrocytes but also in cardiac myocytes and vascular tissues (9). In GD, TSHRAb can bind to these extrathyroidal receptors, potentially contributing to cardiac manifestations like AV block. Vitamin D’s immunomodulatory effects, mediated through its influence on cytokine expression and immune cell activity, could play a role in modulating this autoimmune response (7). Specifically, vitamin D may reduce pro-inflammatory cytokines and enhance regulatory T cells, thereby reducing TSHRAb levels and potentially mitigating TSHR activation in the heart (10, 11).

Independent of its immunomodulatory role, vitamin D also exhibits direct cardioprotective properties. Vitamin D receptors are present in cardiovascular tissues, and vitamin D influences pathways such as the renin-angiotensin-aldosterone system, nitric oxide production, and oxidative stress, potentially improving myocardial function and reducing the risk of conduction abnormalities (12, 13). A randomized clinical trial demonstrated vitamin D supplementation’s positive impact on arterial stiffness and blood pressure in GD patients, further supporting its potential cardiovascular benefits (14).

Despite the theoretical immunomodulatory mechanisms supporting vitamin D’s role in Graves disease, clinical evidence regarding its therapeutic benefit remains controversial and inconsistent. The largest randomized controlled trial to date, the DAGMAR trial, found that vitamin D supplementation (2800 IU daily) did not improve remission rates in Graves disease patients treated with antithyroid drugs and, paradoxically, showed a trend toward worse outcomes (42% failure rate versus 32% in placebo) (15). Similarly, Cho et al. found that while vitamin D supplementation may delay recurrence, it did not significantly prevent Graves disease relapse overall (16). Most concerning, Grove-Laugesen et al. reported that vitamin D supplementation actually impeded restoration of muscle performance and quality of life in newly diagnosed Graves disease patients, causing unfavorable effects on muscle strength recovery compared to placebo (17). However, contrasting evidence exists: Gallo et al. demonstrated that combined selenium and vitamin D supplementation resulted in prompter control of hyperthyroidism and improved quality of life when baseline levels were suboptimal (3). These discrepancies may reflect differences in baseline vitamin D status, dosing regimens, concurrent selenium supplementation, and study populations (10), and demonstrate that the role of vitamin D in Graves disease management remains incompletely understood, with current evidence insufficient to support routine high-dose supplementation in all patients, though further research into specific subpopulations or cardiac manifestations may be warranted.

The combination of methimazole and vitamin D might have synergistic effects. Methimazole, the first-line treatment for hyperthyroidism in pediatric GD, primarily addresses the underlying hyperthyroid state. Vitamin D supplementation, while potentially contributing to thyroid control (3), may offer additional benefits specifically regarding cardiac conduction by reducing the hyperthyroid state, which can exacerbate pre-existing conduction issues. It may also work through its immune-modulating properties reducing thyroid antibodies and thus improving heart block.

Our patient’s avoidance of pacemaker implantation, contrasting with the frequent pacemaker use in Ozcan et al.’s study of thyroid dysfunction-associated AV block (18), highlights the potential for vitamin D to mitigate CHB severity. This suggests vitamin D might offer an alternative or adjunctive approach to traditional management, particularly in cases where less invasive options are preferred. This observation warrants further investigation to identify patients who may benefit most and to clarify the optimal role of vitamin D in this context.

This case report has some limitations. The observational nature prevents establishing causality, and potential confounding factors, such as the variable natural history of AV block in hyperthyroidism (1) and concurrent methimazole therapy, limit the conclusions that can be definitively drawn. This is particularly relevant to the vitamin D intervention, which reflects real-world clinical practice rather than a structured research protocol. Because supplementation was initiated without a baseline vitamin D assessment and the dosage was not part of a controlled trial, it is more difficult to isolate its potential effect from the aforementioned confounders. Consequently, a clear dose-response relationship cannot be determined from this single observation.

## Conclusion

In conclusion, the significant improvement in AV block observed in the case report may be attributed to the combined immunomodulatory and cardioprotective effects of vitamin D. This suggests a potential therapeutic role for vitamin D supplementation in managing cardiac manifestations of GD. Larger studies may assist with establishing a greater understanding of the role of vitamin D in managing cardiac manifestations of GD.

## Patient perspective

Given the development of the CHB surrounding the diagnosis of Graves disease, the family wanted to explore all options to circumvent the placement of a pacemaker. The patient experienced no symptoms of CHB either before or after its resolution. His mother reports that the decision to try vitamin D supplementation stemmed from a recommendation by a family member’s endocrinologist. They felt comfortable trying vitamin D, reasoning that it was unlikely to cause harm and had the potential to help. The family was surprised and delighted by the significant improvement in the CHB following vitamin D initiation, particularly as their initial cardiologist had strongly advocated for pacemaker implantation. Seeking second and third opinions, they found cardiologists who supported a watch-and-wait approach given the patient’s asymptomatic status. The mother believes the vitamin D played a crucial role in modulating her son’s overactive autoimmune response, noting his history of rashes, particularly after his COVID-19 vaccination, and the strong family history of Graves disease. By sharing their experience, the family hopes to raise awareness of the potential benefits of vitamin D and encourage other families to explore all therapeutic options in collaboration with their medical team.

Written informed consent for this case report was obtained from the patient’s mother.

## Data Availability

The original contributions presented in the study are included in the article/supplementary material. Further inquiries can be directed to the corresponding authors.
